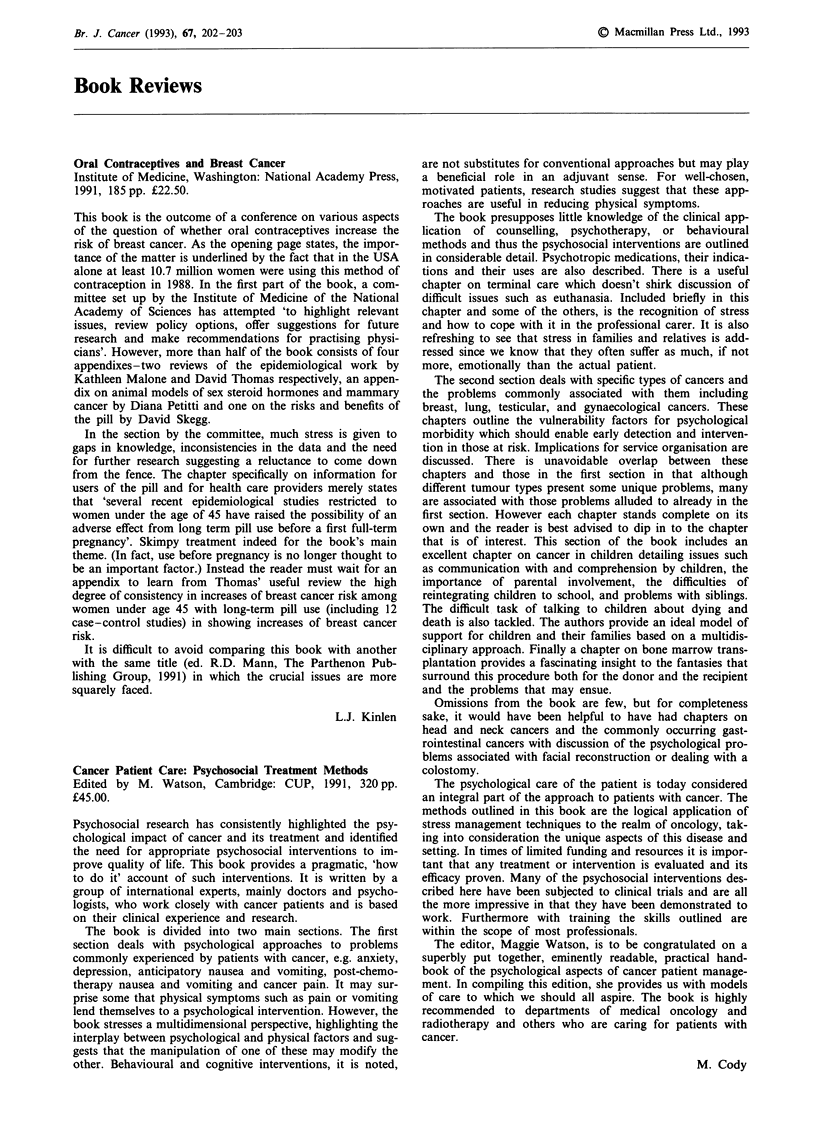# Cancer Patient Care: Psychosocial Treatment Methods

**Published:** 1993-01

**Authors:** M. Cody


**Cancer Patient Care: Psychosocial Treatment Methods**
Edited by M. Watson, Cambridge: CUP, 1991, 320 pp. £45.00.

Psychosocial research has consistently highlighted the psychological impact of cancer and its treatment and identified the need for appropriate psychosocial interventions to improve quality of life. This book provides a pragmatic, 'how to do it' account of such interventions. It is written by a group of international experts, mainly doctors and psychologists, who work closely with cancer patients and is based on their clinical experience and research.

The book is divided into two main sections. The first section deals with psychological approaches to problems commonly experienced by patients with cancer, e.g. anxiety, depression, anticipatory nausea and vomiting, post-chemotherapy nausea and vomiting and cancer pain. It may surprise some that physical symptoms such as pain or vomiting lend themselves to a psychological intervention. However, the book stresses a multidimensional perspective, highlighting the interplay between psychological and physical factors and suggests that the manipulation of one of these may modify the other. Behavioural and cognitive interventions, it is noted, are not substitutes for conventional approaches but may play a beneficial role in an adjuvant sense. For well-chosen, motivated patients, research studies suggest that these approaches are useful in reducing physical symptoms.

The book presupposes little knowledge of the clinical application of counselling, psychotherapy, or behavioural methods and thus the psychosocial interventions are outlined in considerable detail. Psychotropic medications, their indications and their uses are also described. There is a useful chapter on terminal care which doesn't shirk discussion of difficult issues such as euthanasia. Included briefly in this chapter and some of the others, is the recognition of stress and how to cope with it in the professional carer. It is also refreshing to see that stress in families and relatives is addressed since we know that they often suffer as much, if not more, emotionally than the actual patient.

The second section deals with specific types of cancers and the problems commonly associated with them including breast, lung, testicular, and gynaecological cancers. These chapters outline the vulnerability factors for psychological morbidity which should enable early detection and intervention in those at risk. Implications for service organisation are discussed. There is unavoidable overlap between these chapters and those in the first section in that although different tumour types present some unique problems, many are associated with those problems alluded to already in the first section. However each chapter stands complete on its own and the reader is best advised to dip in to the chapter that is of interest. This section of the book includes an excellent chapter on cancer in children detailing issues such as communication with and comprehension by children, the importance of parental involvement, the difficulties of reintegrating children to school, and problems with siblings. The difficult task of talking to children about dying and death is also tackled. The authors provide an ideal model of support for children and their families based on a multidisciplinary approach. Finally a chapter on bone marrow transplantation provides a fascinating insight to the fantasies that surround this procedure both for the donor and the recipient and the problems that may ensue.

Omissions from the book are few, but for completeness sake, it would have been helpful to have had chapters on head and neck cancers and the commonly occurring gastrointestinal cancers with discussion of the psychological problems associated with facial reconstruction or dealing with a colostomy.

The psychological care of the patient is today considered an integral part of the approach to patients with cancer. The methods outlined in this book are the logical application of stress management techniques to the realm of oncology, taking into consideration the unique aspects of this disease and setting. In times of limited funding and resources it is important that any treatment or intervention is evaluated and its efficacy proven. Many of the psychosocial interventions described here have been subjected to clinical trials and are all the more impressive in that they have been demonstrated to work. Furthermore with training the skills outlined are within the scope of most professionals.

The editor, Maggie Watson, is to be congratulated on a superbly put together, eminently readable, practical handbook of the psychological aspects of cancer patient management. In compiling this edition, she provides us with models of care to which we should all aspire. The book is highly recommended to departments of medical oncology and radiotherapy and others who are caring for patients with cancer.

M. Cody